# Genotypic and Phenotypic Relationship among Yield Components in Rice under Tropical Conditions

**DOI:** 10.1155/2018/8936767

**Published:** 2018-07-15

**Authors:** Yusuff Oladosu, M. Y. Rafii, Usman Magaji, Norhani Abdullah, Gous Miah, Samuel C. Chukwu, Ghazali Hussin, Asfaliza Ramli, Isiaka Kareem

**Affiliations:** ^1^Institute of Tropical Agriculture and Food Security, Universiti Putra Malaysia (UPM), 43400 Serdang, Selangor, Malaysia; ^2^Malaysian Agricultural Research and Development Institute (MARDI), 43400 Serdang, Selangor, Malaysia; ^3^Department of Agronomy, University of Ilorin, Nigeria

## Abstract

The associations among yield-related traits and the pattern of influence on rice grain yield were investigated. This evaluation is important to determine the direct and indirect effects of various traits on yield to determine selection criteria for higher grain yield. Fifteen rice genotypes were evaluated under tropical condition at five locations in two planting seasons. The experiment was laid out in a randomized complete block design with three replications across the locations. Data were collected on vegetative and yield components traits. The pooled data based on the analysis of variance revealed that there were significant differences (*p* < 0.001) among the fifteen genotypes for all the characters studied except for panicle length and 100-grain weight. Highly significant and positive correlations at phenotypic level were observed in grain weight per hill (0.796), filled grains per panicle (0.702), panicles per hill (0.632), and tillers per hill (0.712) with yield per hectare, while moderate positive correlations were observed in flag leaf length to width ratio (0.348), days to flowering (0.412), and days to maturity (0.544). By contrast, unfilled grains per panicle (-0.225) and plant height (-0.342) had a negative significant association with yield per hectare. Filled grains per panicle (0.491) exhibited the maximum positive direct effect on yield followed by grain weight per hill (0.449), while unfilled grain per panicle (-0.144) had a negative direct effect. The maximum indirect effect on yield per hectare was recorded by the tillers per hill through the panicles per hill. Therefore, tillers per hill, filled grains per panicle, and grain weight per hill could be used as selection criteria for improving grain yield in rice.

## 1. Introduction

Rice (*Oryza sativa* L.) is an important staple food that constitutes a dominant portion of a world standard diet. In spite of its position among the highly rated cereals, the geometric growth rate of the global population has called for improving the current yield for this extremely important cereal. Thus, several methods have been attempted by scientists to combat this perennial problem. Some researchers have attempted nutritional methods such as physiological methods, breeding, and pest and disease control [1–3]. Among these methods, breeding for high yield traits has been established as the most sustainable method because the traits are heritable. However, yield is a complex trait which is controlled by many factors such as polygene, environment, and genetic variability [4]. Selection for increased grain yield should not be based on yield only because of its complexity and relationship with other yield components. Therefore, other yield-related traits should be taken into consideration.

Path coefficient analysis is a reliable statistical technique used for organising and presenting the cause and effect relationship between the predictor character and response character based on experimental results. It quantifies the interrelationships of different yield components, whether it has a direct influence on the yield or takes another pathway for ultimate effects so that the contribution of each character to yield could be estimated [5]. Path analysis was developed by Wright [6], with a major advantage of portioning the correlation coefficient into direct and indirect effects components. According to Deway and Lu [7], the first component of path analysis is the direct effect of a predictor character upon its response character, while the second component is the indirect effect of a predictor character upon its response character through another predictor character(s).

Path analysis is used in agriculture by plant breeders for identification of characters that can be used as selection criteria for improving grain yield [8]. Except for Bagheri et al. [8], most of the path analyses in rice focusing on grain yield and yield traits considered only a few yield traits. In addition, previous research in path analyses treated yield components traits as the first-order component which resulted in the presence of multicollinearity in path coefficients with values greater than one. The path analysis of grain yield and yield component traits that includes at least second-order components variables in their path diagram is limited in rice. Therefore, the objectives of this study were to acquire and deduce information on the nature of interrelationships between yield and yield component traits among fifteen rice varieties that have been prearranged into first- and second-order predictor character in a path diagram.

Yield is a character that resulted from the association and expression of different yield-related components [4]. Hence, knowledge of the degree of this association through correlation studies can identify traits that could be used as indirect selection criteria for yield or as secondary traits, improving the efficiency of the selection process. If the cause and effect relationship is well understood, then presenting a whole system of the variable in diagram form known as path diagram will be easier [4]. Since correlation coefficient measures only the relationship between two characters and does not actually reveal the relative importance of each trait, this study was conducted to determine the nature of the relationship between grain yield and yield components.

Yield component traits such as plant height, tillers per hill, panicles per hills, panicle length, numbers of filled and unfilled grains per panicle, 100-grain weight, and total grain weight per panicle are important and also essential fundamental task before making any successful breeding program. Path analysis can be used to calculate the quantitative impact on yield of direct or indirect effects caused by one or the other components [9]. Scientists in chilli [4], wheat [10], rapeseed [11], chickpea [5], and cowpea [12] commonly use path coefficient analysis to explain clearly the relations among yield components. Grain yield increment using yield component breeding could be met if the yield component characters are highly heritable and have a positive correlation with the total grain yield. The present investigation was conducted with the following objectives: (i) to find the genetic variability among different plant traits and (ii) to study the interrelationships and influential patterns of some important yield components on rice grain yield by adopting path coefficient analysis tools. The results will then be used as selection criteria for improvement of grain yield in rice under tropical conditions.

## 2. Materials and Methods

### 2.1. Plant Husbandry

Fifteen genotypes comprised of six advanced mutant lines, six mutant varieties from abroad (Vietnam and Bangladesh), and three commercial varieties were used in this study. The commercial varieties of MR219, MR220, and MR253 were developed by the Malaysian Agricultural Research and Development Institute (MARDI) and officially released in 2001. These varieties were the first set of varieties to be developed by means of a direct seeding planting system. The emphasis was on the panicle characteristics, short life cycle (105–111 days), fairly long but strong culms, and being tolerant to blast and bacterial leaf blight. VN121, VN124, and VN001 were developed by Vietnam Atomic Energy Institute (VINATOM), and the main characteristics of these varieties include good ratoon potential and semidwarf stature. Three mutant varieties were obtained from Bangladesh Institute of Nuclear Agriculture (BINA), namely, Binadhan4, Binadhan7, and Iratom. These varieties possess high-tillering capacity but are prone to lodging. Six advanced mutant lines (ML4, ML6, ML9, ML10, ML21, and ML24) were promoted from preliminary studies of ion beam irradiation [13] with an extremely high yielding potential. These mutant lines along with mutant varieties from Vietnam and Bangladesh were selected as a representative of combinations of low and high levels of five important yield determinants. These traits include plant height, days to maturity, tillers per hill, number of panicles, and panicle length. The separation between the low- and high-level genotypes was based on our previous research [14].

The field trials were conducted in five locations in two different cropping seasons in peninsular Malaysia, namely, Kota Sarang Semut, Seberang Perai, Tanjung Karang, Sekinchan, and Serdang. The locations covered a wide range of environmental conditions differing in management practices (farmers' fields versus research field), water system (supplementary irrigation versus rainfed), and temperatures (moderate and warm climate conditions). The locations were chosen to represent major rice producing areas in Malaysia. In each environment, the experiment was laid out in a randomized complete block design with three replications. Plot size was 342 m^2^, with subplot size of 4 m^2^ unit for each genotype in each replication. Optimum date for transplanting at each location was followed according to the farmer's schedule.

Fertilizer application was applied following the recommendation by Malaysian Agricultural Research and Development Institute (MARDI) as nitrogen was applied in form of urea in 30, 55, and 75 days after transplanting at 80, 12, and 20 kg per hectare. NPK fertilizer was also applied in triplicate starting on days 15, 55, and 75 after transplanting at the rate of 140, 107, and 50 kg ha^−1^. Phosphorus (in form of triple superphosphate) was applied at 15 days at the rate of 57kg per ha^−1^ and potassium (applied in form of Murate of potash) at 42kg ha^−1^.

### 2.2. Data Collection

Five hills were randomly selected for each genotype in each replication to record observations for plant height, flag leaf length to width ratio, days to 50% flowering, days to maturity, tillers per hill, panicles per hill, panicle length, filled grains per panicle, unfilled grains per panicle, 100-grain weight, and grain weight per hill. Yield in ton per hectare (ton ha^−1^) was estimated from the weight of threshed grains from all panicles in 1.5 × 1.5 m^2^, excluding border rows.

### 2.3. Coefficient Analysis

The genotypic and phenotypic correlation coefficient estimates were carried out using SAS, version 9.4 (SAS Institute Inc., Cary, NC, USA). Association of the various characters with yield per hectare was worked out at genotypic and phenotypic levels as described by Kashiani and Saleh [15]. The phenotypic correlations were further partitioned into components of direct and indirect effects using path coefficient analysis according to the method given by Wright [6]. The path coefficients were calculated following Usman et al. [4], where sets of simultaneous equations were arranged in matrix notation that reveals the relationships between correlations and path coefficients as shown in the equation below. In these equations, *r* represents the phenotypic correlations values between variables, while *P* values are the direct effects of one variable upon another and *r*ij*P*ij values are the indirect effects. Each observation is defined according to their serial number.

  1 = Plant height
  2 = Flag leaf length to width ratio
  3 = Days to flowering
  4 = Days to maturity
  5 = Tillers per hill
  6 = Panicles per hill
  7 = Panicle lengths
  8 = filled grains per panicle
  9 = Unfilled grains per panicle
  10 = 100-grain weight
  11 = Grain weight per hill
  12 = Yield in t ha^−1^.

*Effects of Vegetative and Yield Component Variables on Yield per Hectare*

 
*r*_112=_*P*_112+_*r*_12_*P*_212+_*r*_13_*P*_312+_*r*_14_*P*_412+_*r*_15_*P*_512+_*r*_16_*P*_612+_*r*_17_*P*_712+_*r*_18_*P*_812+_*r*_19_*P*_912+_*r*_110_*P*_1012+_*r*_111_*P*_1112_
 
*r*_212=_*r*_21_*P*_112+_*P*_212+_*r*_23_*P*_312+_*r*_24_*P*_412+_*r*_25_*P*_512+_*r*_26_*P*_612+_*r*_27_*P*_712+_*r*_28_*P*_812+_*r*_29_*P*_912+_*r*_210_*P*_1012+_*r*_211_*P*_1112_
 
*r*_312=_*r*_31_*P*_112+_*r*_32_*P*_212+_*P*_312+_*r*_34_*P*_412+_*r*_35_*P*_512+_*r*_36_*P*_612+_*r*_37_*P*_712+_*r*_38_*P*_812+_*r*_39_*P*_912+_*r*_310_*P*_1012+_*r*_311_*P*_1112_
 
*r*_412=_*r*_41_*P*_112+_*r*_42_*P*_212+_*r*_43_*P*_312+_*P*_412+_*r*_45_*P*_512+_*r*_46_*P*_612+_*r*_47_*P*_712+_*r*_48_*P*_812+_*r*_49_*P*_912+_*r*_410_*P*_1012+_*r*_411_*P*_1112_
 
*r*_512=_*r*_51_*P*_112+_*r*_52_*P*_212+_*r*_53_*P*_312+_*r*_54_*P*_412+_*P*_512+_*r*_56_*P*_612+_*r*_57_*P*_712+_*r*_58_*P*_812+_*r*_59_*P*_912+_*r*_510_*P*_1012+_*r*_511_*P*_1112_
 
*r*_612=_*r*_61_*P*_112+_*r*_62_*P*_212+_*r*_63_*P*_312+_*r*_64_*P*_412+_*r*_65_*P*_512+_*P*_612+_*r*_67_*P*_712+_*r*_68_*P*_812+_*r*_69_*P*_912+_*r*_610_*P*_1012+_*r*_611_*P*_1112_
 
*r*_712=_*r*_71_*P*_112+_*r*_72_*P*_212+_*r*_73_*P*_312+_*r*_74_*P*_412+_*r*_75_*P*_512+_*r*_76_*P*_612+_*P*_712+_*r*_78_*P*_812+_*r*_79_*P*_912+_*r*_710_*P*_1012+_*r*_711_*P*_1112_
 
*r*_812=_*r*_81_*P*_112+_*r*_82_*P*_212+_*r*_83_*P*_312+_*r*_84_*P*_412+_*r*_85_*P*_512+_*r*_86_*P*_612+_*r*_87_*P*_712+_*P*_812+_*r*_89_*P*_912+_*r*_810_*P*_1012+_*r*_811_*P*_1112_
 
*r*_912=_*r*_91_*P*_112+_*r*_92_*P*_212+_*r*_93_*P*_312+_*r*_94_*P*_412+_*r*_95_*P*_512+_*r*_96_*P*_612+_*r*_97_*P*_712+_*r*_98_*P*_812+_*P*_912+_*r*_910_*P*_1012+_*r*_911_*P*_1112_
 
*r*_1012=_*r*_101_*P*_112+_*r*_102_*P*_212+_*r*_103_*P*_312+_*r*_104_*P*_412+_*r*_105_*P*_512+_*r*_106_*P*_612+_*r*_107_*P*_712+_*r*_108_*P*_812+_*r*_109_*P*_912+_*P*_1012+_*r*_1011_*P*_1112_
 
*r*_1112=_*r*_111_*P*_112+_*r*_112_*P*_212+_*r*_113_*P*_312+_*r*_114_*P*_412+_*r*_115_*P*_512+_*r*_116_*P*_612+_*r*_117_*P*_712+_*r*_118_*P*_812+_*r*_119_*P*_912+_*r*_1110_*P*_1012+_*P*_1112_.

The studied traits were further subdivided into two-stage relations: first-order components and second-order components. The first-order components include plant height, flag leaf length to width ratio, days to flowering, days to maturity, and tillers per hill. The second-order components are panicles per hill, panicle lengths, filled grains per panicle, unfilled grains per panicle, 100-grain weight, and grain weight per hill. The cause and effect relationships between the two components were worked out using additional simultaneous equations arranged in matrix notation as indicated in the equations below. 

*Effects of First-Order Components on the Panicles per Hill, Panicle Lengths, Grains per Panicle, Unfilled Grains per Panicle, 100-Grain Weight, and Weight per Hill*

 
***Panicles per Hill***
 
*r*_16  =_  *P*_16  +_ * r*_12_*P*_26  +_ * r*_13_*P*_36  +_ * r*_14_*P*_46  +_ * r*_15_*P*_56_
 
*r*_26  =_ * r*_21_*P*_16  +_  *P*_26  +_ * r*_23_*P*_36  +_ * r*_24_*P*_46  +_ * r*_25_*P*_56_
 
*r*_36  =_ * r*_31_*P*_16  +_ * r*_32_*P*_26  +_ * P*_36 + _*r*_34_*P*_46  +_ * r*_35_*P*_56_
 
*r*_46  =_ * r*_41_*P*_16  +_ * r*_42_*P*_26  +_ * r*_43_*P*_36  +_  *P*_46  +_ * r*_45_*P*_56_
 
*r*_56=_*r*_51_*P*_16  +_ * r*_52_*P*_26  +_ * r*_53_*P*_36  +_ * r*_54_*P*_46  +_ * P*_56_.
 
***Panicle Lengths***
 
*r*_17  =_  *P*_17  +_ * r*_12_*P*_27  +_ * r*_13_*P*_37  +_ * r*_14_*P*_47  +_ * r*_15_*P*_57_
 
*r*_27  =_ * r*_21_*P*_17  +_  *P*_27  +_ * r*_23_*P*_37  +_ * r*_24_*P*_47  +_ * r*_25_*P*_57_
 
*r*_37  =_ * r*_31_*P*_17  +_ * r*_32_*P*_27  +_  *P*_37  +_ * r*_34_*P*_47  +_ * r*_35_*P*_57_
 
*r*_47  =_ * r*_41_*P*_17  +_ * r*_42_*P*_27  +_ * r*_43_*P*_37  +_  *P*_47  +_ * r*_45_*P*_57_
 
*r*_57  =_ * r*_51_*P*_17  +_ * r*_52_*P*_27  +_ * r*_53_*P*_37  +_ * r*_54_*P*_47  +_ * P*_57_.

 
***Grains per Panicle***
 
*r*_18  =_  *P*_18  +_ * r*_12_*P*_28  +_ * r*_13_*P*_38  +_ * r*_14_*P*_48  +_ * r*_15_*P*_58_
 
*r*_28  =_ * r*_21_*P*_18  +_  *P*_28  +_ * r*_23_*P*_38  +_ * r*_24_*P*_48  +_ * r*_25_*P*_58_
 
*r*_38  =_ * r*_31_*P*_18  +_ * r*_32_*P*_28  +_  *P*_38  +_ * r*_34_*P*_48  +_ * r*_35_*P*_58_
 
*r*_48  =_ * r*_41_*P*_18  +_ * r*_42_*P*_28  +_ * r*_43_*P*_38  +_  *P*_48  +_ * r*_45_*P*_58_
 
*r*_58  =_ * r*_51_*P*_18  +_ * r*_52_*P*_28  +_ * r*_53_*P*_38  +_ * r*_54_*P*_48  +_ * P*_58_.
 
***Unfilled Grains per Panicle***
 
*r*_19  =_  *P*_19  +_ * r*_12_*P*_29  +_ * r*_13_*P*_39  +_ * r*_14_*P*_49  +_ * r*_15_*P*_59_
 
*r*_29  =_ * r*_21_*P*_19  +_  *P*_29  +_ * r*_23_*P*_39  +_ * r*_24_*P*_49  +_ * r*_25_*P*_59_
 
*r*_39  =_ * r*_31_*P*_19  +_ * r*_32_*P*_29  +_  *P*_39  +_ * r*_34_*P*_49  +_ * r*_35_*P*_59_
 
*r*_49  =_ * r*_41_*P*_19  +_ * r*_42_*P*_29  +_ * r*_43_*P*_39  +_  *P*_49  +_ * r*_45_*P*_59_
 
*r*_59  =_ * r*_51_*P*_19  +_ * r*_52_*P*_29  +_ * r*_53_*P*_39  +_ * r*_54_*P*_49  +_ * P*_59_.

 
***Unfilled Grains per Panicle***
 
*r*_19  =_  *P*_19  +_ * r*_12_*P*_29  +_ * r*_13_*P*_39  +_ * r*_14_*P*_49  +_ * r*_15_*P*_59_
 
*r*_29  =_ * r*_21_*P*_19  +_  *P*_29  +_ * r*_23_*P*_39  +_ * r*_24_*P*_49  +_ * r*_25_*P*_59_
 
*r*_39  =_ * r*_31_*P*_19  +_ * r*_32_*P*_29  +_  *P*_39  +_ * r*_34_*P*_49  +_ * r*_35_*P*_59_
 
*r*_49  =_ * r*_41_*P*_19  +_ * r*_42_*P*_29  +_ * r*_43_*P*_39  +_  *P*_49  +_ * r*_45_*P*_59_
 
*r*_59  =_ * r*_51_*P*_19  +_ * r*_52_*P*_29  +_ * r*_53_*P*_39  +_ * r*_54_*P*_49  +_ * P*_59_.
 
***100-Grain Weight***
 
*r*_110  =_  *P*_110  +_ * r*_12_*P*_210  +_ * r*_13_*P*_310  +_ * r*_14_*P*_410  +_ * r*_15_*P*_510_
 
*r*_210  =_ * r*_21_*P*_110  +_  *P*_210  +_ * r*_23_*P*_310  +_ * r*_24_*P*_410  +_ * r*_25_*P*_510_
 
*r*_310  =_ * r*_31_*P*_110  +_ * r*_32_*P*_210  +_  *P*_310  +_ * r*_34_*P*_410  +_ * r*_35_*P*_510_
 
*r*_410  =_ * r*_41_*P*_110  +_ * r*_42_*P*_210  +_ * r*_43_*P*_310  +_  *P*_410  +_ * r*_45_*P*_510_
 
*r*_510  =_ * r*_51_*P*_110  +_ * r*_52_*P*_210  +_ * r*_53_*P*_310  +_ * r*_54_*P*_410  +_ * P*_510_.

 
***100-Grain Weight***
 
*r*_110  =_  *P*_110  +_ * r*_12_*P*_210  +_ * r*_13_*P*_310  +_ * r*_14_*P*_410  +_ * r*_15_*P*_510_
 
*r*_210  =_ * r*_21_*P*_110  +_  *P*_210  +_ * r*_23_*P*_310  +_ * r*_24_*P*_410  +_ * r*_25_*P*_510_
 
*r*_310  =_ * r*_31_*P*_110  +_ * r*_32_*P*_210  +_  *P*_310  +_ * r*_34_*P*_410  +_ * r*_35_*P*_510_
 
*r*_410  =_ * r*_41_*P*_110  +_ * r*_42_*P*_210  +_ * r*_43_*P*_310  +_  *P*_410  +_ * r*_45_*P*_510_
 
*r*_510  =_ * r*_51_*P*_110  +_ * r*_52_*P*_210  +_ * r*_53_*P*_310  +_ * r*_54_*P*_410  +_ * P*_510_.
 
***Grain Weight per Hill***
*r*_111  =_ *P*_111  +_ * r*_12_*P*_211  +_ * r*_13_*P*_311  +_ * r*_14_*P*_411  +_ * r*_15_*P*_511_
 
*r*_211  =_ * r*_21_*P*_111  +_  *P*_211  +_ * r*_23_*P*_311  +_ * r*_24_*P*_411  +_ * r*_25_*P*_511_
 
*r*_311  =_ * r*_31_*P*_111  +_ * r*_32_*P*_211  +_  *P*_311  +_ * r*_34_*P*_411  +_ * r*_35_*P*_511_
 
*r*_411  =_ * r*_41_*P*_111  +_ * r*_42_*P*_211  +_ * r*_43_*P*_311  +_  *P*_411  +_ * r*_45_*P*_511_
 
*r*_511  =_ * r*_51_*P*_111  +_ * r*_52_*P*_211  +_ * r*_53_*P*_311  +_ * r*_54_*P*_411  +_ * P*_51_.

*Effects of Second-Order Components on Yield per Hectare*

 
*r*_612  =_  *P*_612  +_ * r*_67_*P*_712  +_ * r*_68_*P*_812  +_ * r*_69_*P*_912  +_ * r*_610_*P*_1012  +_ * r*_611_*P*_1112_
 
*r*_712  =_ * r*_76_*P*_612  +_  *P*_712  +_ * r*_78_*P*_812  +_ * r*_79_*P*_912  +_ * r*_710_*P*_1012  +_ * r*_711_*P*_1112_
 
*r*_812  =_ * r*_86_*P*_612  +_ * r*_87_*P*_712  +_  *P*_812  +_ * r*_89_*P*_912  +_ * r*_810_*P*_1012  +_ * r*_811_*P*_1112_
 
*r*_912  =_ * r*_96_*P*_612  +_ * r*_97_*P*_712  +_ * r*_98_*P*_812  +_  *P*_912  +_ * r*_910_*P*_1012  +_ * r*_911_*P*_1112_
 
*r*_1012  =_ * r*_106_*P*_612  +_ * r*_107_*P*_712  +_ * r*_108_*P*_812  +_ * r*_109_*P*_912  +_  *P*_1012  +_ * r*_1011_*P*_1112_
 
*r*_1112  =_ * r*_116_*P*_612  +_ * r*_117_*P*_712  +_ * r*_118_*P*_812  +_ * r*_119_*P*_912  +_ * r*_1110_*P*_1012  +_*P*_1112_.

## 3. Results and Discussion

### 3.1. Analysis of Variance

The data presented in Table 1 represent the pooled analysis of variance for the vegetative, yield, and yield component traits for all the genotypes across the entire locations. There was a significant difference among the genotypes, environment, and the genotype by environment interaction as indicated in Table 1. The observed significant differences indicate the presence of considerable amount of genetic variation that exists between the genotypes evaluated. The genetic variability observed in any breeding materials shows the brighter chances of producing desirable traits of plant and perhaps can be used in heterosis breeding [13]. The differences showed by the genotypes could be because of their genetic background and their origination from different source. In this direction, several reports have been published on phenotypic variation among rice genotypes. Pandey et al. [16] reported highly significant differences among 40 rice accessions with the use of 12 quantitative characters. Similarly, using 20 morphological characters, Rao [17] discovered 95% differences among five rice populations.

### 3.2. Phenotypic and Genotypic Correlation

In this study, the phenotypic and genotypic correlation coefficient of vegetative, yield, and yield component are separated for a clear understanding as shown in Table 2. The proc corr in SAS program provides the r-values and the test of its significance as present in Table 2. The result showed that yield per hectare had a positive correlation with other traits except for unfilled grain per panicle and plant height. Grain yield trait does not exist in isolation but rather as a result of an association with other traits that form a complex relationship that ultimately affects the yield. This association may be either positive or negative. The r-value for Karl Pearson's correlation coefficient helps in identification of an association between two distinct traits, although it does not measure the magnitude of association but it does give the idea of the relationship. For the correlation coefficient interpretations, Ratner [18] gives a standard accepted guideline. The r-value of 0, +1, and -1 indicates no linear relationship, a perfect positive linear relationship, and negative linear relationship, respectively. The values that range from 0 to 0.3, 0.3 to 0.7, and 0.7 to 1 indicate a low, moderate, and strong positive linear relationships, respectively, while the values that range from 0 to -0.3, -0.3 to -0.7, and -0.7 to -1 indicate a low, moderate, and strong negative linear relationships, respectively.

The correlation coefficient of phenotypic characters ranges from 0.026 to 0.818 while the genotypic character correlation coefficient ranges from 0.04 to 0.919. This indicates that there is higher magnitude at the genotypic level in most cases as compared with the corresponding phenotypic level. The yield per hectare shows a strong, positive, and highly significant correlation with grain weight per hill, tillers per hill, filled grains per panicle, and panicles per hill, respectively. Selection based on these four components is effective due to their equal contribution towards grain yield increment.

### 3.3. Direct and Indirect Effects of Vegetative Traits on Yield per Hectare

**Figure 1:**
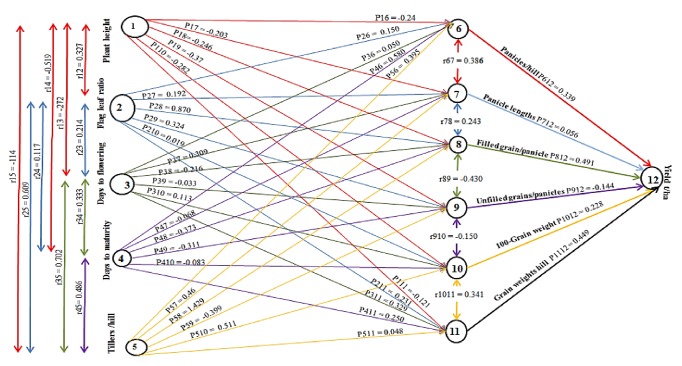
Path diagram and coefficients of factors on the influence of first-order on second-order components and the latter on yield per hectare, *P*ij values are the direct effects, and* r*ij values are the correlation coefficients.** Note: **in the path diagram, the single arrowed lines represent direct influence while the doubled-arrowed lines indicate a mutual association.

Grain yield is considered as the artefact of all the contributory traits (plant height, flag leaf length to width ratio, days to flowering, days to maturity, tillers per hill, panicles per hill, panicle length, total numbers of grains per panicle, unfilled grains per panicle, and weight per hill), and the correlation coefficient of these contributory factors with final yield are partitioned into direct and indirect effects as presented in Figure 1 and Table 3. These allow the separation of direct and indirect effect through other traits by allotting the correlations for better interpretation of cause and effect relationship [6].

The present study revealed a significant interrelationship among various vegetative and yield components. These traits define the limitation to yield per hectare and the component characters through the direct and indirect effects as a result of interrelationships between them. Therefore, the use of path coefficient analysis investigates the direct and indirect relationships among the component characters through the partitioning of correlation coefficients [6]. The path analysis revealed that the maximum direct effect on yield per hectare was exerted by grain weight per hill followed by tillers per hill and filled grains per panicle; however, the maximum indirect effect on yield per hectare was recorded by the tillers per hill through the panicles per hill (Figure 1). These characters can be used to develop an optimally reliable selection index for realizing improvements in grain yield in rice.

### 3.4. Two-Stage Relations

The vegetative traits were grouped as the first-order component which includes the following: plant height, flag leaf length to width ratio, days to flowering, days to maturity, and tillers per hill. The second-order component was regarded as yield component traits which were regarded as the principal yield determining factors in rice, and these comprise panicles per hill, panicle length, filled grains per panicle, unfilled grains per panicle, 100-grain weight, and grain weight per hill. The interrelation between these two components is presented in Tables 4 and 5.

### 3.5. Effect of First-Order Component Relation on Second-Order Component

The path of the influence of first-order component on panicles per hill (Table 4) revealed that only plant height had a negative correlation while other parameters showed a positive relationship. The days to maturity had the highest value of direct correlation with panicles per hill followed by tillers per hill. The path analysis of first-order component with panicle length revealed a negative direct effect on plant height and day to maturity while other parameters such as flag leaf length to width ratio, days to flowering, and tillers per hill show a positive direct effect (Table 4). The interrelationships of plant height, days to flowering, and day to maturity with the grains per panicle showed a negative direct effect while tillers per hill and flag leaf length to width ratio showed a positive direct effect (Table 4). It was reported by Rahman et al. [19] that the flag leaf area was directly related to the yield components. Also, flag leaves play a significant role in enhancing rice yield because these leaves remain the only source of assimilating during the grain filling stage [19]. The larger the flag leaf area is, the more the solar interception and photosynthate productions are, provided that all other factors of production are not limiting. Moreover, tillers per hill had positive and the highest direct effect (0.928) on filled grains per panicle. This is because the tillers per hill play a significant role in determining the yield of the rice grain since it is directly related to panicle number that will be produced per unit ground area. Fewer tillers result in fewer panicles; excess tillers cause high tiller abortions, small panicles, poor grain filling, and reduction in grain yield [14]. The path analysis relationship of first-order component with the unfilled grains per panicle showed that only flag leaf length to width ratio had a positive direct effect while other parameters show a negative direct effect (Table 4). Also, the influence of first-order component on 100-grain weight revealed that plant height and days to maturity showed a negative direct effect while flag leaf length to width ratio, days to flowering, and tillers per hill had a positive and direct effect. The relationship of plant height, flag leaf length to width ratio, days to flowering, days to maturity, and tillers per hill relation with grain weight per hill show positive and direct effect except for plant height which had a negative and direct effect (Table 4). As indirectly pointed out earlier, rice yield is indirectly related to its height. This is due to sink competition for the limited photosynthates produced by limited sources. So what will be used for yield increase will be unnecessarily used for somatic cell enlargement that results in dense vegetative growth and enhanced height.

### 3.6. Second-Order Component on Yield

The path analysis of second-order component on yield was presented in Table 5. It is revealed that filled grains per panicle (0.491) exhibited the maximum positive direct effect on yield. This was supported by Seyoum et al. [20] who have stated that cultivars with higher grains per panicle showed higher grain yield in rice. Although number of grains per panicle depends on tillers per hill and panicle per hill, its suitability as selection criteria in crop improvement program is mainly dependent on the percentage of filled grains. Grain weight per hill (0.449) also had a positive direct effect on yield followed panicle per hill (0.339). Number of panicles per plant is an important trait contributing to total grain yield as it has a positive relationship to yield. Generally, cultivars with higher panicle number are desirable towards crop improvement. Panicle length (0.056) and 100-grain weight (0.228) also had a positive direct effect but weak relationship while unfilled grain per panicle (-0.144) had a negative direct effect. It could be recalled that grain weight per hill showed the highest genotypic correlation (0.871) with the yield. This strong correlation was as a result of a high positive direct effect on yield. Many previous research works conducted on rice showed similar result [20–22]. On the other hand, the highest positive and direct effect has been reported on panicle number [23], days to maturity [24], and spikelet fertility [25]. It could be concluded that the grains per panicle and weight per hill should be given priority during selection in rice improvement program because of their influence on grain yield.

## 4. Conclusion

The critical analysis of path coefficient analysis and partitioning of correlation reveals that tillers per hill and grain weight per hill possessed positive direct effect and positive association with yield per hectare. Selection for the improvement of grain yield can be efficient if it is based on tillers per hill and grain weight per hill because of their contribution directly towards grain yield. Breeders in these areas should, therefore, develop early maturing genotypes focusing on tillers per hill and grain weight per hill for improving the grain yield per plant in both rainfed and irrigated area.

## Figures and Tables

**Table 1:** Mean squares of vegetative traits, yield, and yield component of the rice genotype.

Traits	Reps in Env (df=20)	Genotypes (G) (df=14)	Environments (E) (df=9)	G × E (df=126)	Error (df=280)
PH	127.30^*∗∗*^	3213.43^*∗∗*^	6221.85^*∗∗*^	83.83^*∗∗*^	10.72
FLWR	25.38^*∗∗*^	655.24^*∗∗*^	362.44^*∗∗*^	100.65^*∗∗*^	5.33
DF	20.15^*∗∗*^	2155.86^*∗∗*^	669.93^*∗∗*^	99.65^*∗∗*^	1.97
DM	19.66^*∗∗*^	2247.16^*∗∗*^	1188.56^*∗∗*^	103.99^*∗∗*^	5.58
TPH	41.19^*∗∗*^	83.56^*∗∗*^	720.43^*∗∗*^	48.54^*∗∗*^	9.59
PPH	36.06^*∗∗*^	56.82^*∗∗*^	407.1^*∗∗*^	27.05^*∗∗*^	10.82
PL	11.7^ns^	23.3^*∗*^	63.6^*∗*^	4.9^*∗*^	0.88
FGP	666.56^*∗∗*^	9646.28^*∗∗*^	54389.4^*∗∗*^	1735.15^*∗∗*^	196.624
UFGP	116.42^ns^	384.89^*∗∗*^	1293.34^*∗∗*^	151.72^*∗∗*^	80.12
100-GW	0.13^*∗*^	0.13^*∗∗*^	0.07^*∗*^	0.06^*∗*^	0.02
GWH	163.97^ns^	587.41^*∗∗*^	24754.5^*∗∗*^	254.61^*∗∗*^	151.9
YLD	1.45^*∗*^	19.50^*∗∗*^	84.09^*∗∗*^	4.93^*∗∗*^	2.38

^*∗*^Significant at *p* ≤ 0.05, ^*∗∗*^highly significant at *p* ≤ 0.01, ns: nonsignificant *p* > 0.05, df: degree of freedom, PH: plant height, FLWR: flag leaf length to width ratio, DF: days to flowering, DM: days to maturity, TPH: tillers per hill, PPH: panicles per hill, PL: panicle length, FGP: Filled grains per panicle, UFGP: unfilled grains per panicle, GWH: grain weight per hill, 100-GW: 100-grain weight, and YLD: yield in t/ha.

**Table 2:** Estimates of correlation coefficients at phenotypic (**indicated in bold**) and genotypic levels among 12 traits in rice genotypes.

		FLR	DF	DM	TH	PPH	PL	FGP	UFGP	100-GW	GWH	YLD
PH	**P**	**0.327** ^**∗**^	**-0.272** ^**ns**^	**-0.519** ^**∗∗**^	**-0.114** ^**ns**^	**-0.334** ^**∗**^	**-0.194** ^**ns**^	**-0.366** ^**∗∗**^	**0.285** ^**∗**^	**-0.321** ^**∗**^	**-0.270** ^**ns**^	**-0.342** ^**∗**^
	G	0.487^**ns**^	-0.401^**ns**^	-0.521^**ns**^	-0.191^**ns**^	-0.386^**ns**^	-0.211^**ns**^	-0.428^**∗**^	0.148^**ns**^	-0.332^**∗**^	-0.281^**ns**^	-0.484^**∗∗**^
FLR	**P**		**0.214** ^**ns**^	**0.117** ^**ns**^	**0.609** ^**∗∗**^	**0.462** ^**∗∗**^	**0.211** ^**ns**^	**0.060** ^**ns**^	**0.026** ^**ns**^	**0.252** ^**ns**^	**0.321** ^**∗**^	**0.348** ^**∗∗**^
	G		0.277^**∗**^	0.028^**ns**^	0.628^**∗**^	0.756^**∗∗**^	0.145^**ns**^	0.123^**∗**^	0.053^**∗**^	0.249^**ns**^	0.234^**∗∗**^	0.521^**∗**^
DF	**P**			**0.333** ^**∗**^	**0.702** ^**∗∗**^	**0.559** ^**∗∗**^	**0.414** ^**∗∗**^	**0.593** ^**∗∗**^	**-0.337** ^**∗**^	**0.525** ^**∗∗**^	**0.528** ^**∗∗**^	**0.412** ^**∗∗**^
	G			-0.099^**ns**^	0.752^**∗**^	NA	0.332^**ns**^	0.713^**∗∗**^	-0.109^**∗**^	0.545^**ns**^	0.352^**∗**^	0.692^**∗∗**^
DM	**P**				**0.486** ^**∗∗**^	**0.818** ^**∗∗**^	**0.184** ^**ns**^	**0.303** ^**∗**^	**-0.459** ^**∗∗**^	**0.351** ^**∗∗**^	**0.473** ^**∗∗**^	**0.544** ^**∗∗**^
	G				0.647^**∗**^	0.856^**∗∗**^	0.065^**ns**^	0.355^**ns**^	-0.342^**ns**^	0.37^**ns**^	0.351^**∗**^	0.792^**∗**^
TH	**P**					**0.806** ^**∗∗**^	**0.369** ^**∗∗**^	**0.735** ^**∗∗**^	**-0.372** ^**∗∗**^	**0.593** ^**∗∗**^	**0.555** ^**∗∗**^	**0.712** ^**∗∗**^
	G					NA	NA	0.85^**∗∗**^	NA	0.606^**∗∗**^	NA	0.821^**∗∗**^
PPH	**P**						**0.386** ^**∗∗**^	**0.489** ^**∗∗**^	**-0.467** ^**∗∗**^	**0.594** ^**∗∗**^	**0.542** ^**∗∗**^	**0.632** ^**∗∗**^
	G						0.25^**∗∗**^	0.605^**∗∗**^	-0.28^**∗∗**^	0.61^**∗∗**^	0.338^**∗**^	0.819^**∗**^
PL	**P**							**0.243** ^**ns**^	**0.257** ^**ns**^	**0.817** ^**∗∗**^	**0.253** ^**ns**^	**0.257** ^**ns**^
	G							0.538^**∗∗**^	0.355^**∗**^	0.809^**∗∗**^	-0.027^ns^	0.395^**∗**^
FGP	**P**								**-0.430** ^**ns**^	**0.549** ^**∗∗**^	**0.506** ^**∗∗**^	**0.702** ^**∗∗**^
	G								-0.597^**∗**^	0.506^**∗∗**^	0.561^**∗**^	0.639^**∗∗**^
UFGP	**P**									**-0.150** ^**ns**^	**-0.114** ^**ns**^	**-0.225** ^**ns**^
	G									-0.131^**∗**^	NA	0.04^**∗**^
100-GW	**P**										**0.341** ^**∗**^	**0.404** ^**∗∗**^
	G										0.393^**∗**^	0.582^**∗∗**^
GWH	**P**											**0.796** ^**∗∗**^
	G											0.871^**∗**^

PH: plant height, FLWR: flag leaf length to width ratio, DF: days to flowering, DM: days to maturity, TH: tillers per hill, PPH: panicles per hill, PL: panicle length, FGP: Filled grains per panicle, UFGP: Unfilled grains per panicle, GWH: grain weight per hill, 100-GW: 100-grain weight, YLD: yield in t/ha, P: phenotypic correlation, and G: genotypic correlation.

**Table 3:** Phenotypic path analysis of the direct (diagonal) and indirect effects of eleven traits on yield per hectare in the rice genotypes.

	PH	FLR	DF	DM	TH	PPH	PL	FGP	UFGP	100-GW	GWH
PH	**-0.143**	-0.047	0.039	0.074	0.016	0.048	0.028	-0.041	0.052	0.046	0.039
FLR	0.022	**0.067**	0.014	0.008	0.041	0.031	0.014	0.002	0.004	0.017	0.021
DF	0.117	-0.092	**-0.429**	-0.143	-0.301	-0.240	-0.178	0.145	-0.255	-0.225	-0.227
DM	-0.081	0.018	0.052	**0.157**	0.076	0.128	0.029	-0.072	0.047	0.055	0.074
TH	-0.056	0.296	0.341	0.236	**0.487**	0.392	0.180	-0.181	0.358	0.289	0.270
PPH	0.047	-0.065	-0.078	-0.115	-0.113	**-0.140**	-0.054	0.066	-0.069	-0.083	-0.076
PL	-0.058	0.063	0.123	0.055	0.110	0.115	**0.298**	0.077	0.073	0.243	0.075
FGP	-0.142	0.023	0.231	0.118	0.286	0.190	0.095	**-0.167**	0.389	0.213	0.197
UFGP	-0.012	-0.001	0.015	0.020	0.016	0.020	-0.011	-0.044	**0.019**	0.007	0.005
100-GW	0.109	-0.086	-0.178	-0.119	-0.202	-0.202	-0.277	0.051	-0.186	**-0.340**	-0.116
GWH	-0.144	0.171	0.282	0.252	0.296	0.289	0.135	-0.061	0.270	0.182	**0.534**
YLD	-0.342^*∗*^	0.348^*∗∗*^	0.412^*∗∗*^	0.544^*∗∗*^	0.712^*∗∗*^	0.632^*∗∗*^	0.257^ns^	0.702^*∗∗*^	-0.225^ns^	0.404^*∗∗*^	0.796^*∗∗*^

PH: plant height, FLWR: flag leaf length to width ratio, DF: days to flowering, DM: days to maturity, TH: tillers per hill, PPH: panicles per hill, PL: panicle length, FGP: filled grains per panicle, UFGP: Unfilled grains per panicle, 100-GW: 100-grain weight, GWH: grain weight per hill, and YLD: yield in t/ha.

**Table 4:** Relationship between first order and the second order.

	Variable	PH	FLR	DF	DM	TPH
Panicle per hill	PH	**-0.024**	-0.008	0.006	0.012	0.003
	FLR	0.049	**0.150**	0.032	0.018	0.092
	DF	-0.014	0.011	**0.050**	0.017	0.035
	DM	-0.301	0.068	0.193	0.**580**	0.282
	NT	-0.045	0.240	0.277	0.192	**0.395**
	PPH	-0.334^*∗*^	0.462^*∗∗*^	0.559^*∗∗*^	0.818^*∗∗*^	0.806^*∗∗*^

Panicle length	PH	**-0.203**	-0.066	0.055	0.105	0.023
	FLR	0.063	**0.192**	0.041	0.022	0.117
	DF	-0.084	0.066	**0.309**	0.103	0.217
	DM	0.035	-0.008	-0.023	**-0.068**	-0.033
	NT	-0.005	0.028	0.032	0.022	**0.046**
	PL	-0.194^ns^	0.211^ns^	0.414^*∗∗*^	0.184^ns^	0.369^*∗∗*^

Filled grains per panicle	PH	**-0.246**	-0.081	0.067	0.128	0.029
	FLR	-0.209	**0.870**	-0.137	-0.075	-0.089
	DF	0.059	-0.046	**-0.216**	-0.072	-0.029
	DM	0.194	-0.044	-0.124	**-0.373**	-0.104
	NT	-0.163	-0.639	1.003	0.694	**0.928**
	TNG	-0.366^*∗∗*^	0.060^ns^	0.593^*∗∗*^	0.303^*∗*^	0.735^*∗∗*^

Unfilled grains per panicle	PH	**-0.037**	-0.012	0.010	0.019	0.004
	FLR	0.106	**0.324**	0.069	0.038	0.197
	DF	0.009	-0.007	**-0.033**	-0.011	-0.023
	DM	0.162	-0.036	-0.104	**-0.311**	-0.151
	NT	0.046	-0.243	-0.280	-0.194	**-0.399**
	NUFGP	0.285^ns^	0.026^ns^	-0.337^*∗*^	-0.459^*∗∗*^	-0.372^*∗∗*^

100-grain weight	PH	**-0.282**	-0.092	0.077	0.146	0.032
	FLR	0.006	**0.019**	0.004	0.002	0.011
	DF	-0.031	0.024	**0.113**	0.038	0.080
	DM	0.043	-0.010	-0.028	**-0.083**	-0.040
	NT	-0.058	0.311	0.358	0.248	**0.511**
	GW	-0.322^*∗*^	0.252^ns^	0.525^*∗∗*^	0.351^*∗*^	0.593^*∗∗*^

Grain weight per hill	PH	**-0.121**	-0.039	0.033	0.063	0.014
	FLR	0.076	**0.231**	0.049	0.027	0.141
	DF	-0.090	0.070	**0.329**	0.110	0.231
	DM	-0.130	0.029	0.083	**0.250**	0.122
	NT	-0.005	0.029	0.034	0.023	**0.048**
	TGW	-0.270^ns^	0.320^*∗*^	0.528^*∗∗*^	0.473^*∗∗*^	0.555^*∗∗*^

PH: plant height, FLWR: flag leaf length to width ratio, DF: days to flowering, DM: days to maturity, and TH: tillers per hill.

**Table 5:** Second-order component on yield per plant.

Variable	PPH	PL	FGH	UFGP	100-GW	GWH
PPH	**0.339**	0.131	0.166	-0.158	0.103	0.184
PL	0.022	**0.056**	0.013	0.268	0.046	0.014
FGH	0.24	0.119	**0.491**	-0.211	0.24	0.249
UFGP	-0.067	0.037	-0.062	**-0.144**	-0.367	-0.016
100-GW	-0.145	-0.2	-0.134	0.071	**0.228**	-0.084
GWH	0.243	0.114	0.228	-0.051	0.154	**0.449**
YLD	0.632^*∗∗*^	0.257^ns^	0.702^*∗∗*^	-0.225^ns^	0.404^*∗∗*^	0.796^*∗∗*^

PPH: panicles per hill, PL: panicle length, FGP: filled grains per panicle, UFGP: unfilled grains per panicle, GWH: grain weight per hill, 100-GW: 100-grain weight, and YLD: yield in t/ha.

## Data Availability

All data and information used to support the findings of this study are included within the article's tables and figure.
